# Draft Genome Sequences for Bacteria Associated with Root Nodules of Alnus incana in New England

**DOI:** 10.1128/mra.00914-22

**Published:** 2022-11-07

**Authors:** Kelsey Mercurio, Joseph Sevigny, Céline Pesce, W. Kelley Thomas, Louis S. Tisa

**Affiliations:** a Department of Molecular, Cellular, and Biomedical Sciences, University of New Hampshire, Durham, New Hampshire, USA; b Hubbard Center for Genome Studies, University of New Hampshire, Durham, New Hampshire, USA; University of Arizona

## Abstract

Nine bacterial strains isolated from the root nodules of Alnus incana were sequenced to determine their potential roles in plant health. The selected bacterial isolates belonged to the genera *Bacillus, Herbaspirillum*, *Pantoea*, *Paenibacillus*, and *Rothia*. Here, we report the draft genome sequences.

## ANNOUNCEMENT

Besides hosting endosymbionts, root nodules have other occupants or plant endophytes that appear to assist plant growth and health (1–5). Using a culture-independent approach, we elucidated the nodule microbiome of Casuarina glauca (1) and have extended this study to Alnus incana found in New England. We previously reported the genome sequences of 10 bacterial isolates obtained in 2018 (6). Here, we continue to isolate more endophytes of alder nodules.

In September and November 2019, root nodule samples were collected from A. incana found by Adams Point at Jackson’s laboratory in Durham, New Hampshire. The root nodules were surface sterilized with hydrogen peroxide and rinsed several times with sterile distilled water. The nodule was cut into a fine powder with a sterilized razor, and dilutions were plated onto Czapek (7) and R2A (8) media. About 77 isolates were initially obtained, purified, and propagated on either Czapek or R2A media. These isolates were incubated overnight in their respective isolation medium (Table 1), and genomic DNA (gDNA) was extracted by cetyltrimethylammonium bromide (CTAB) DNA extraction protocol (9). RNA was removed by RNase treatment. The quality and quantity of the gDNA were verified by a Thermo Scientific NanoDrop. Nine isolates were chosen for whole-genome sequencing analysis to provide insight into their plant-microbe interactions, including potential plant growth-promoting activity.

**TABLE 1:** Genome characteristics

Bacterial species	Isolate^*a*^	GenBank accession no.	SRA accession no.	No. of reads	No. of contigs	Avg. coverage (×)	Genome assembly size (bp)	*N*_50_ contig size (bp)	No. of CDSs^*b*^	G+C content (%)	No. of rRNAs	No. of tRNAs
Bacillus velezensis	alder76	JAIUDC000000000	SRX12008554	3,356,518	34	209.7	4,341,411	341,132	4,334	45.7	15	80
Bacillus velezensis	alder77	JAIUDB000000000	SRX12008555	3,171,512	32	198.2	4,340,492	341,132	4,332	45.7	15	80
Bacillus velezensis	alder71	JAIUDD000000000	SRX12008553	3,179,306	34	198.7	4,340,599	341,132	4,337	45.7	15	80
*Herbaspirillum* sp.	alder98	JAIUCY000000000	SRX12008550	4,825,154	26	301.5	5,342,531	530,052	4,764	62.3	3	53
*Paenibacillus* sp.	alder61	JAIUDG000000000	SRX12008549	4,198,488	63	262.4	6,112,889	244,801	5,371	53.0	25	79
*Pantoea* sp.	alder81	JAIUDA000000000	SRX12008556	6,011,958	35	375.7	5,643,466	534,706	5,206	53.4	12	71
*Pantoea* sp.	alder70	JAIUDE000000000	SRX12008552	4,283,982	81	267.7	5,648,269	140,384	5,244	53.4	14	70
*Pantoea* sp.	alder69	JAIUDF000000000	SRX12008551	4,000,000	42	250	5,648,814	534,706	5,219	53.4	12	71
Rothia kristinae	alder54	JAIUDH000000000	SRX12008548	4,144,358	18	259	2,344,478	266,588	2,050	71.9	4	47

a Bacteria were isolated on Czapek (alder54, alder61, alder69, and alder 70) and R2A (alder71, alder76, alder77, alder81, and alder98) media.

b CDSs, coding DNA sequences.

Whole-genome sequencing was performed at the Hubbard Center for Genome Studies (University of New Hampshire, Durham, NH) using Illumina technology techniques (10). A paired-end library was constructed using a Nextera DNA library preparation kit (Illumina, San Diego, CA) and sequenced on an Illumina NovoSeq instrument to produce 250-bp paired-end reads. The total numbers of reads for all 9 strains are listed in Table 1. The Illumina sequence data were trimmed by Trimmomatic version 0.36 (11). TruSeq adapters were trimmed with an allowance of two mismatches. Leading and trailing bases below the quality of 3 were trimmed. The read was then scanned with a sliding window of 4 bp and trimmed if the average quality dropped below 30. Finally, reads were dropped if the length was less than 36 bp. Trimmed sequencing reads were assembled using SPAdes version 3.15.2 (12) with the default settings. Default parameters were used for all software unless otherwise specified. The assembled genomes were annotated via the NCBI Prokaryotic Genome Annotation Pipeline (PGAP) (13). The assembly metrics and annotation features are given in Table 1.

The identities of the strains were determined by a whole genome-based taxonomic analysis via the Type (Strain) Genome Server (TYGS) platform (14) (https://tygs.dsmz.de), including digital DNA-DNA hybridization (dDDH) values (15). The type-based species clustering using a 70% dDDH radius around each of the type strains was used as previously described (16), while subspecies clustering was done using a 79% dDDH threshold as previously introduced (17). Among the nine isolates, the three Bacillus velezensis strains are identical to each other, and the type strain and *Paenibacillus* sp. alder61 isolates were characterized as belonging to Paenibacillus faecis. Although three *Pantoea* isolates are identical to each other, they represent a potential new species, as does the *Herbaspirillum* isolate.

### Data availability.

The draft genome sequences of these bacterial strains have been deposited in GenBank under the accession numbers listed in Table 1. Both the assembly and raw reads are available at DDBJ/ENA/GenBank under BioProject accession number PRJNA748777.
